# Distribution and budget of ^137^Cs in the China Seas

**DOI:** 10.1038/s41598-020-65280-x

**Published:** 2020-05-29

**Authors:** Junwen Wu, Xiyu Xiao, Jiang Sun

**Affiliations:** 1 Institute of Marine Sciences, Guangdong Provincial Key Laboratory of Marine Biotechnology, Shantou University, Shantou, 515063 China; 2 Southern Marine Science and Engineering Guangdong Laboratory, Guangzhou, 511458 China

**Keywords:** Environmental impact, Ocean sciences, Marine chemistry

## Abstract

Cesium–137 is one of the most abundant anthropogenic radionuclides released by atmospheric nuclear testing and nuclear accidents, and accordingly it may significantly impact the health of humans and marine environmental eco–systems. Documenting the distribution and inventory of ^137^Cs is thus a crucial task. In this study, we collected a large number of datasets with field observations of ^137^Cs in the China Seas, in order to provide an in–depth understanding of ^137^Cs budgets and distributions. The activity and inventory of ^137^Cs in China Seas’ sediments showed large spatial variations, related to the ^137^Cs source, sedimentation rates and the mineral composition of sediments. The ^137^Cs concentration in sediments decreased with distance from the shore, generally tracing the distribution of sedimentation rates. High ^137^Cs inventories in the water column indicated a high solubility and long mean residence times. The mean residence times of ^137^Cs in the China Seas were determined to be 45.6 ± 3.8 years for the South China Sea (SCS), 36.8 ± 3.1 years for the East China Sea (ECS), and 12.0 ± 1.0 years for the Yellow Sea (YS). A ^137^Cs mass balance suggests that oceanic input from the north Pacific is the dominant ^137^Cs source to the China Seas, contributing about 96.9% of this substance. Furthermore, the bulk of ^137^Cs remains dissolved in the SCS water column, while ^137^Cs is mostly deposited to the sediments of the ECS and the YS. This new compilation of the activity level and inventory of ^137^Cs help to establish background levels for future ^137^Cs studies in the China Seas.

## Introduction

The fission product ^137^Cs (t_1/2_ = 30.17 years) has been introduced into the marine environment by nuclear bombs, nuclear accidents, and nuclear power plants since the 1940s. The estimated ^137^Cs inventory of the global ocean is ~603 PBq (1 PBq=10^15^ Bq), accounting for over 60% of the total global fallout^1^. Additionally, ^137^Cs has high radiotoxicity and is easily absorbed by muscular tissues. Therefore, the environmental impact of ^137^Cs has become of great concern to the society since the Fukushima Dai–ichi Nuclear Power Plant (FDNPP) accident, which occurred in 2011^2^.

^137^Cs is an excellent tracer that is widely applied to study environmental processes. For example, ^137^Cs is used to study currents, circulation and water mass movement in the oceanic environment^3,4^. The profile of ^137^Cs in sediment cores is typically used to reconstruct its depositional history^5,6^. In the terrestrial environment, ^137^Cs is an effective tracer in soil erosion studies^7–10^. In past decades, ^137^Cs has been widely investigated in order to evaluate its environmental behavior and impact^3,4,11–14^.

Accurate ^137^Cs measurement is very important for ^137^Cs related studies. The analytical tool conventionally used to measure ^137^Cs activity in environmental samples is γ–spectrometry. However, γ–spectrometry has several notable disadvantages, such as requiring a large–volume sample (e.g., ~100 L seawater), laborious pretreatment and a long counting time^15^. Therefore, compared to ordinary marine measurements, such as temperature, salinity and nutrients, very limited marine ^137^Cs datasets are available.

**Figure 1:**
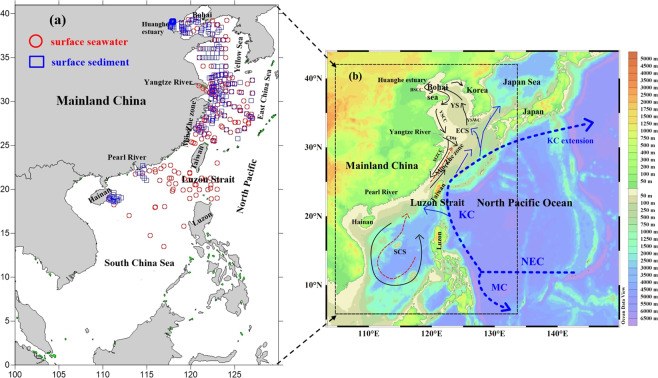
Maps of: (**a**) seawater and sediment sampling sites in the China Seas. The red circles and blue squares represent surface seawater and surface sediment sample collection sites, respectively. The sampling locations of surface seawater and surface sediment are cited from previous studies (East China Sea^5,13,24–26,31,44,45,49^; South China Sea^6,12,13,28,30,39,76^; Yellow Sea^26,27,29^). (**b**) a schematic chart of the China Seas surface current: the Kuroshio, and the North Equatorial Current (NEC), represented by the blue dotted lines. In the South China Sea (SCS), the red dashed and black solid lines indicate the anticyclonic gyre in summer and the cyclonic gyre in winter, respectively. In the East China Sea (ECS), the red dashed and black solid lines represent the coastal currents in summer and winter, respectively. We modified this pattern of ocean circluation according to Hu *et al*.^77^. Acronyms include: BSCC: Bohai Sea Coastal Current, CDW: Changjiang Diluted Water, KC: Kuroshio Current, MZCC: Min–Zhe Coastal Current, MC: Mindanao Current, NEC: North Equatoral Current, TWC: Taiwan Warm Current, YSWC: Yellow Sea Warm Current. Figure (a) was prepared with Golden software surfer 10.0 (10.0 version, https://www.goldensoftware.com). Figure (b) was drawn using the free software Ocean Data View (ODV 5.1.2) (Schlitzer, R., Ocean Data View, https://odv.awi.de, 2018).

The present study focuses on the China Seas, which are located in the western North Pacific (WNP) and include the East China Sea (ECS), South China Sea (SCS), Yellow Sea (YS) and Bohai Sea. The China Seas interact intensely with the surrounding lands and are influenced by nutrients and terrestrial particulate matters discharged from large rivers: e.g., the Yangtze River, the Pearl River, and the Yellow River. Additionally, the China Seas have very complicated circulation patterns due to the influence of the East Asian Monsoon. For example, the surface current in the SCS is an anti–cyclonic gyre in summer while the weak southwest monsoon prevails^16,17^. In contrast, its circulation pattern is a cyclonic gyre in winter while the strong northeast monsoon prevails^16,17^. The Taiwan Current flows northward in summer, and the ECS coastal current flows southward in winter^18^ (Fig. 1). Additionally, the Kuroshio Current, a bifurcation of the North Equatorial Current (NEC), is an important control on the dynamic exchange between the China Seas and the WNP^19^. Across the Luzon Strait, the Kuroshio current exhibits a seasonal pattern, with a weaker intrusion into the SCS in summer than in winter^20^. The Kuroshio flows northeastward along Taiwan’s eastern coast throughout the year, with a branch entering the ECS at the northeast corner of Taiwan while the mainstream continues to flow northeast to the eastern Japanese coast^21^ (Fig. 1). In the YS, the Yellow Sea Cold Water Mass, which is the cold water located below the seasonal thermocline from spring to autumn, and tides, which are mixed diurnal or semi–diurnal^22,23^, are the most important hydrographic features^22^.

To our knowledge, reports on the spatiotemporal distribution of ^137^Cs and its influencing factors in the China Seas as a whole are scarce, with most publications focusing on localized areas. For instance, the activity level and distribution of ^137^Cs in ECS seawater were investigated after the FDNPP accident^24,25^. Zhang *et al*.^26^ discussed the source and budget of ^137^Cs in the ECS covering a small area based on a limited ^137^Cs dataset. Hao *et al*.^27^ investigated the activity level of ^137^Cs in coastal seawater of the Bohai Sea. In the SCS, Yamada *et al*.^12^ and Zhou *et al*.^28^ investigated the horizontal and vertical distribution of ^137^Cs in the water column. Additionally, ^137^Cs in sediments of the ECS was investigated to calculate the apparent sedimentation rates^5^, while Zhuang *et al*.^29^ studied the impact of tides on the distribution of ^137^Cs in Bohai Sea sediment; in the SCS, ^137^Cs in sediments was used to trace the transport of terrestrial particles^30^. However, the limited study areas present just a glimpse of ^137^Cs behavior, which may lead to biased interpretations of ^137^Cs behavior in the China Seas. Expanded ^137^Cs datasets including both seawater and sediment are rarely reported, which limits our ability to fully understand the distribution of ^137^Cs and discuss its important influencing factors in the China Seas.

The objective of this study is to provide detailed insight into the distribution and budget of ^137^Cs in the China Seas, by compiling expanded ^137^Cs datasets that include both seawater and sediments and investigating the factors influencing them. This will also improve our general understanding of the fate of ^137^Cs in marine environments. Furthermore, information about the activity and budget of ^137^Cs also contributes important background information that can be used for future risk assessment in the China Seas, and for the study of ^137^Cs biogeochemistry in marginal seas. Finally, the prospect of future ^137^Cs studies in the China Seas is discussed.

## Methods

### Data source and treatment

We reviewed over 400 datasets of ^137^Cs in the China Seas covering the past 30 years, which include surface measurements and profiles in both sediment and seawater. Detailed sample information is shown in the Supplementary Information (SI). These ^137^Cs datasets were mainly extracted from bibliometric databases such as the Web of Science, Google Scholar and Scopus, using the keywords “East China Sea, South China Sea, and Yellow Sea” combined with the primary keyword “^137^Cs”. For ease of presentation and comparison, all ^137^Cs datasets are uniformly decay–corrected to January 1, 2020.

## Results and discussion

### Distribution of ^137^Cs in the China Seas

#### Seawater

##### Horizontal distribution of ^137^Cs

**Figure 2:**
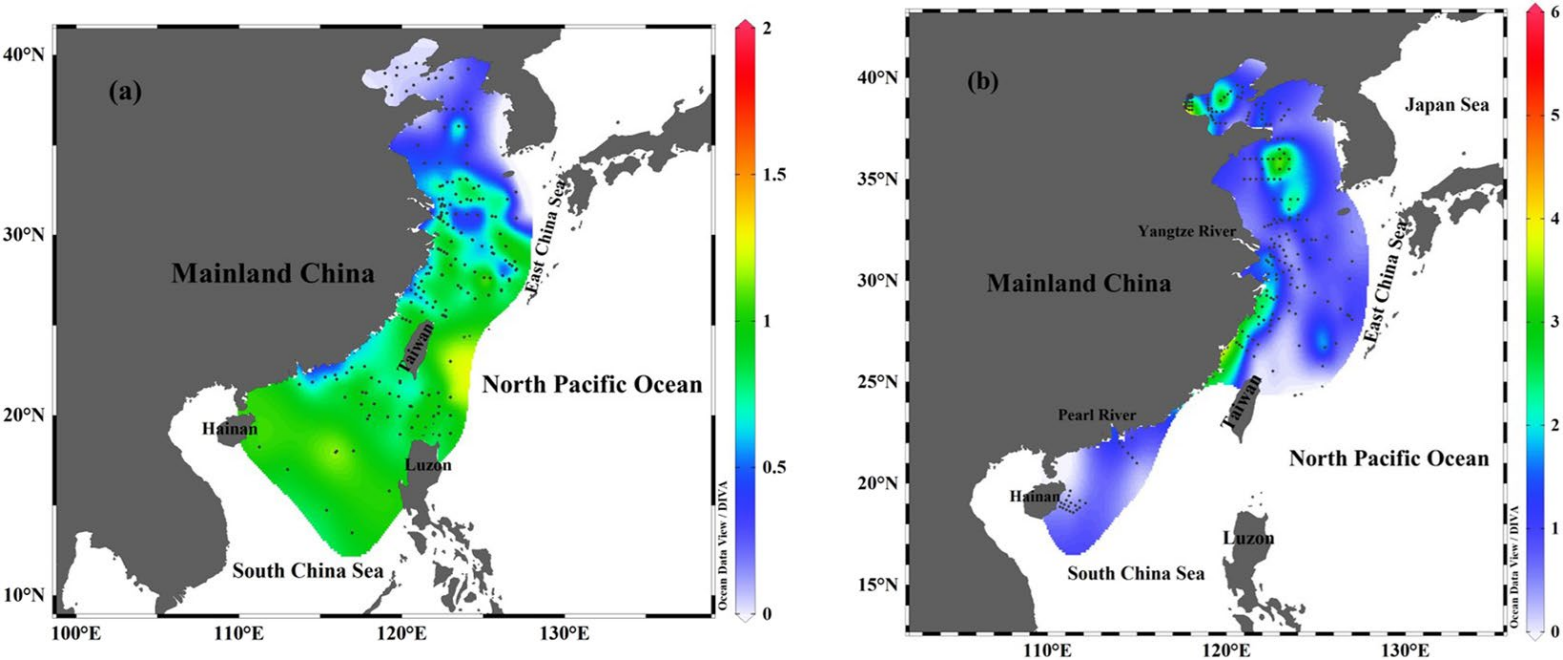
The lateral distribution of ^137^Cs activity (Bq m^−3^) in seawater (**a**) and in sediment (Bq kg^−1^) (**b**) of the China Seas. ^137^Cs data are cited from previous studies (East China Sea^5,13,24–26,31,44,45,49^; South China Sea^6,12,13,28,30,71^; Yellow Sea^14,26,27,29^). This figure was drawn using the free software Ocean Data View (ODV 5.1.2) (Schlitzer, R., Ocean Data View, https://odv.awi.de, 2018).

**Figure 3:**
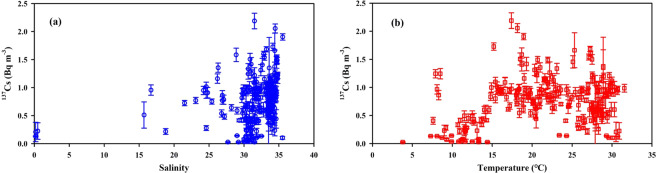
The relationships between ^137^Cs activity and the salinity (**a**) and temperature (**b**) of surface seawater in the China Seas (data soured from previous studies^12,13,25–28,31^). This figure was prepared with Sigma–Plot professional 10.0 software.

Over 200 datasets containing information on surface seawater ^137^Cs in the China Seas were combined^12,13,25–28,31^. The ^137^Cs activity in surface seawater of the China Seas varied from 0.03 to 1.80 Bq m^−3^, averaging 0.74 ± 0.34 Bq m^−3^ (Fig. 2). Spatially, the ^137^Cs activity in the SCS surface seawater varied from 0.47 to 1.80 Bq m^−3^, averaging 0.92 ± 0.28 Bq m^−3^. In the ECS, surface seawater ^137^Cs activity ranged from 0.10–1.37 Bq m^−3^, with a mean of 0.71 ± 0.27 Bq m^−3^. In the YS, ^137^Cs activity in surface water ranged from 0.03 to 0.90 Bq m^−3^, averaging 0.20 ± 0.20 Bq m^−3^. As shown in Fig. 2a, the horizontal distribution of ^137^Cs activities displayed three significant features: (1) the ^137^Cs activity in the Kuroshio was higher than in the China Seas (i.e., a declining trend from the SCS basin to the Luzon Strait); (2) the ^137^Cs activity gradually decreased from south to north along the China Seas with respect to latitude (i.e., the ^137^Cs activity in the SCS was comparable to that in the ECS, but higher than in the YS); (3) the ^137^Cs activity in the nearshore and the estuary (e.g., Yangtze River mouth) was lower than in the marginal sea. Overall, these patterns of ^137^Cs activity are influenced by the source, transportation and scavenging of ^137^Cs. Potential ^137^Cs sources in the China Seas are from the WNP, riverine inputs, and the deposition of global fallout. Outside of the Luzon Strait, the higher ^137^Cs activity in Kuroshio water compared to the China Seas indicates that the outer WNP is a potential ^137^Cs source to the China Seas. The ^137^Cs input flux from the WNP will be discussed in detail later. As shown in Fig. 3a, the low ^137^Cs activities appeared in low salinity zones, while the high ^137^Cs activities occurred in high salinity zones. This also indicates that the source of ^137^Cs potentially originates from the WNP and is derived from the Kuroshio current.

Fluvial input is an additional ^137^Cs source to the China Seas, due to the abundant detrital materials delivered by the rivers of China (e.g., the Pearl River, Yangtze River and Yellow River) that may carry ^137^Cs^26,32^. For example, the Yangtze River discharges a terrestrial particle flux of about 1.3 × 10^8^ tons/year into the ECS^33^. However, terrestrial inputs have differing effects on ^137^Cs cycling. On the one hand, ^137^Cs in the river watershed originating from global fallout is bound to soil and sediment particles, which are discharged into the China Seas due to soil erosion. Based on the “fingerprint” of Pu, Xu *et al*.^34^ calculated the global fallout ^239+240^Pu contribution to the ECS using a two end–member mixing model. They found that riverine input accounts for ~80% of ECS inventories and direct deposition accounts for ~20%. Here, we assumed that ^137^Cs and ^239+240^Pu originating from global fallout have a similar chemical behavior^35^ (the riverine input flux of ^137^Cs is also calculated later in discussion). On the other hand, abundant particles facilitate rapid scavenging of ^137^Cs from the ECS water column, resulting in the lower ^137^Cs activity observed in the Yangtze River mouth compared to the shelf or basin of the ECS. This is also consistent with the ^137^Cs showing a stronger particle affinity in the low salinity or freshwater zones because the high salinity in seawater causes desorption of ^137^Cs^36,37^. The ^137^Cs activity in the China Seas gradually decreases with increasing latitude (e.g., from mid–latitude (SCS and ECS) to high–latitude (YS)), which is inconsistent with the ^137^Cs deposition flux of global fallout, namely, the deposition flux of ^137^Cs in high–latitudes is higher than that observed in low–latitudes since the atmospheric nuclear weapons testing in the early 1960s was mostly conducted in high latitude zones^3^. Such a difference is possibly caused by the different oceanic regimes. The SCS and ECS are deeper and most ^137^Cs is preserved in the water column. The shallower YS experiences strong hydrodynamic conditions (e.g., storms, stronger winds, and intensified waves), which result in the resuspension of particles and vigorous mixing. Therefore, the ^137^Cs in the YS is more readily scavenged from the water column and then deposited in the sediment.

Additionally, the relationship between temperature and ^137^Cs was examined based on a large number of field observations. We found no correlation (Fig. 3b), suggesting that the seasonal variation of ^137^Cs in the China Seas was minor. This may be related to the long half–life and residence time of ^137^Cs in the China Seas (see discussion below)^38^.

##### Vertical distribution of ^137^Cs

**Figure 4:**
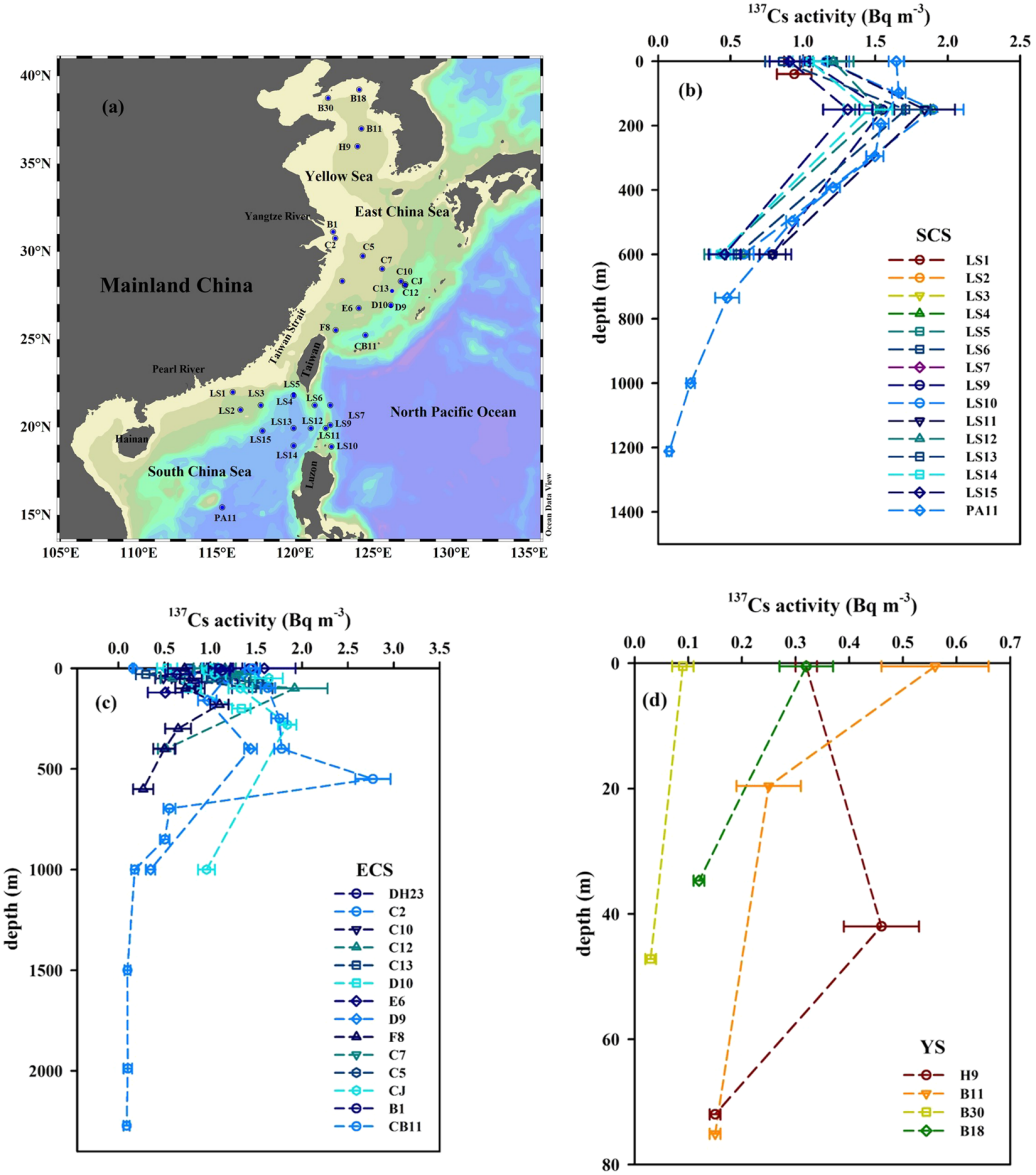
Sampling sites of ^137^Cs in the water column of the China Seas (**a**) and its vertical distributions in the South China Sea (**b**), East China Sea (**c**) and Yellow Sea (**d**). The water column ^137^Cs data are cited from previous studies^24–26,28,31,39^. This figure was prepared with the free software Ocean Data View (ODV 5.1.2) (Schlitzer, R., Ocean Data View, https://odv.awi.de, 2018) (a) and Sigma–Plot professional 10.0 software (b–d).

The ^137^Cs activities in 33 water column locations of the China Seas were collected in previous studies^24–26,28,31,39^, and their vertical distributions are plotted in Fig. 4.

The vertical distributions of ^137^Cs activity in the SCS are shown in Fig. 4b, displaying an initial increasing tendency with water depth and a maximum in the subsurface, followed by a more gradual decrease. This profile distribution of ^137^Cs agrees with those obtained elsewhere^40^. For example, in the Pacific Ocean and the Atlantic Ocean, the vertical pattern of ^137^Cs gradually increases from surface to subsurface (~150 m), which is followed by a slower decrease^3,11,41,42^. The high activity level of ^137^Cs in the SCS subsurface seawater (~150 m) is potentially related to the input of Kuroshio (i.e., the SCS seawater exchanging with the Kuroshio through the Luzon Strait) and the difference of particle removal across this depth layer (namely, higher removal above and lower removal below). The vertical distribution of suspended matter indicates suspended particulate matter (SPM) from the SCS shelf through lateral transport was noticeable in this depth range, which would enhance ^137^Cs scavenging^43^. In the middle deep layer, the concentration of SPM was low, and regeneration should be the dominant process because of SPM microbial decomposition during downward transport^43^.

In the ECS, the vertical distribution of ^137^Cs activity was similar to the pattern in the SCS, namely, a remarkable maximum usually occurred in the subsurface at depths ranging from 100–600 m (Fig. 4c). Wide depth range of the ECS subsurface maxima is potentially due to the relatively wide spatial coverage of the collected samples. For example, the spatial coverage of sampling extends from the estuary to the shelf and the basin. In the YS, the vertical pattern of ^137^Cs activity had no consistent distribution (Fig. 4d), which was related to the tidal effect (semi–diurnal and diurnal tides) and the sampling locations in the coastal area. Nevertheless, they overall showed the ^137^Cs activity in the YS surface seawater was higher than that in the bottom water.

#### Sediment

##### Horizontal distribution of ^137^Cs

**Figure 5:**
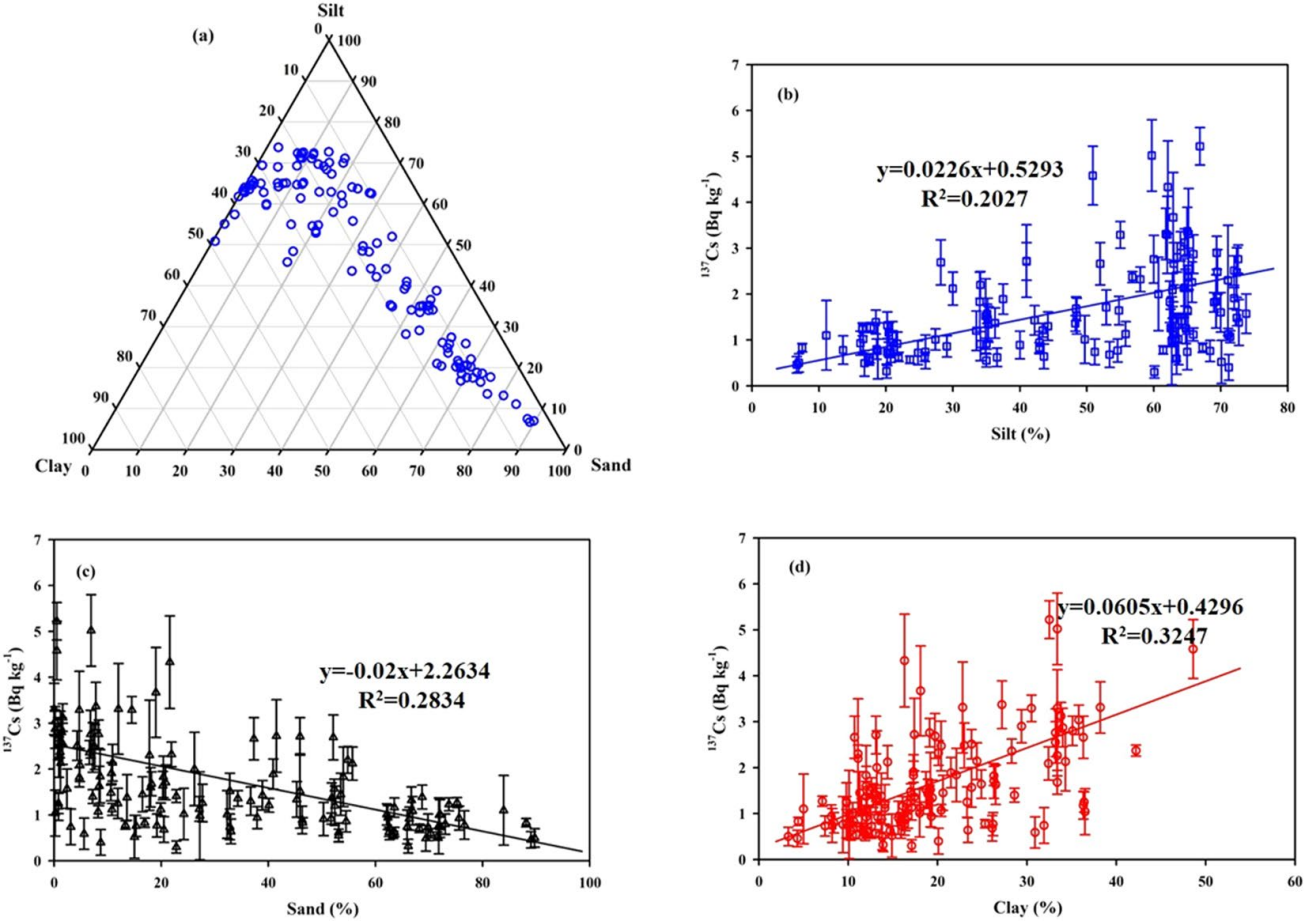
Mineral composition of surface sediment in the China Seas (**a**) and its relationship with ^137^Cs activity (**b–d**). Data sourced from the previous studies^14,26,30,45,49,50^. This figure was prepared with Sigma–Plot professional 10.0 software.

**Figure 6:**
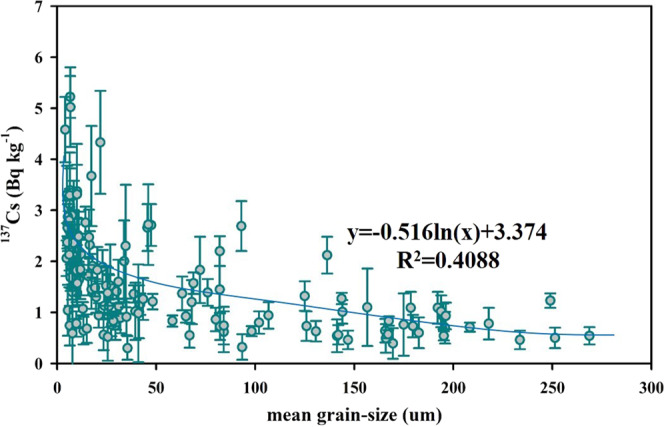
The relationship between mean grain–size and ^137^Cs activity in surface sediments of the China Seas. Data sourced from the previous studies^14,30,45,49,50^. This figure was prepared with Sigma–Plot professional 10.0 software.

The lateral distribution of ^137^Cs activity in the China Seas sediment is plotted in Fig. 2b based on compilation of over 205 datasets. The ^137^Cs activity of surface sediment in the China Seas varies widely from 0.06 to 5.55 Bq kg^−1^, averaging 1.44 ± 1.07 Bq kg^−1^^14,25,29,31,44,45^. Overall, the horizontal distribution of ^137^Cs activity decreases from nearshore to offshore in the China Seas sediment. This pattern is mainly controlled by the ^137^Cs source, transportation, sedimentation rate, mineral composition and particle size. Given that ^137^Cs is mainly bound to riverine particles, it follows the deposition patterns of riverine particles. For example, high ^137^Cs activity is observed in the Min–Zhe coastal zone and the northwest corner of Taiwan in the ECS. The high ^137^Cs activity observed in the Min–Zhe coastal zone was caused by a large abundance of terrestrial particle–bound ^137^Cs. The terrestrial particulate matters entrained by Changjiang diluted water (CDW) in winter, and the resuspended sediments generated by the typhoons in summer, eventually join the northeastwardly Kuroshio in the northern Taiwan Strait^46^. Therefore, the terrestrial particulate matters and resuspended sediments create favorable conditions for the resulting high ^137^Cs activity in the northwest corner of Taiwan. In contrast, the distribution of ^137^Cs activity in the northern SCS shelf shows a decrease from nearshore to offshore, and high ^137^Cs activity is observed on both sides of the Pearl River Estuary (PRE). In the northern SCS, the Pearl River plume disperses southwestward in winter, but northeastward in summer^47^. The terrestrial particulate matter carried by the Pearl River plume is preferentially deposited along the dispersing direction of the plume and favors quick ^137^Cs removal^26^. It is worth noting that we cannot present the spatial distribution with a high resolution because of the limited number of samples available. Finally, the distribution of ^137^Cs activity in the YS is likely controlled by tides, ocean current, fluvial input, and resuspension. In the Yellow River estuary and the central YS, high ^137^Cs activity was observed, while low ^137^Cs activity was observed in the western YS. The Bohai sea, a semi–enclosed bay, connects Yellow River discharge upstream with the YS downstream. The estuarine circulation is mainly influenced by riverine influx and tides^48^. The semi–diurnal and diurnal tides around the Bohai bay are favorable for the resuspension of ^137^Cs in the shallow area. High ^137^Cs activity in the central YS is potentially related to the mineral composition of sediment (i.e., clay, silt and sand)^26^. Further examination of the relationship between ^137^Cs activity and mineral composition/mean size of sediment in the China Seas is shown in Fig. 5. The sediments are mainly composed of silt (2–63 μm), sand (>63 μm) and clay (<2 μm), of which range from 6.7%–73.8% of silt (averaging 46.9%±20.1%, n = 148), 0.2%–89.7% of sand (averaging 34.1%±21.9%, n = 148), and 3.3%–48.6% of clay (averaging 19.0% ± 9.5%, n = 148)^26,30,45,49,50^. By analyzing a large number of field observation data, the ^137^Cs activity showed linear positive correlations with the content of clay (R^2^ = 0.3247) and silt (R^2^ = 0.2027), and a negative correlation with the content of sand (R^2^ = 0.2834) (Fig. 5b–d). The mineral compositions of clay and silt mainly include illite, chlorite, kaolinite and smectite, which more easily adsorbs ^137^Cs. In contrast, the mineral composition of sand is mainly silicon dioxide, which is unfavorable for ^137^Cs adsorbed on the particles. Laboratory experiment also suggested the adsorption of ^137^Cs in sediment depends on the grain size and have reported this type of empirical relationship^51^. Here, the relationship between ^137^Cs activity and grain size was examined based on in–situ data, indicating the ^137^Cs activity exponentially decreased with increasing particle size (Fig. 6). The ^137^Cs is easily adsorbed and accumulated in the finer particles compared to the coarser particles^51^. Walling and Woodward (1992)^52^ suggested the ^137^Cs activity in the finer fractions of Jackmoor Brook catchment soil (Devon, UK) was several times higher than those in the coarser fractions. Indeed, the mineral composition and grain size in sediments of the China Seas has a significant influence on the distribution of ^137^Cs activity. For example, high percentage of clay minerals (>25%) and small grain size in sediments of the Min–Zhe coastal zone corresponds to high ^137^Cs activity^26^. Similar behavior is observed in the Yellow River estuary (percentage of clay >25%) and the central YS (percentage of clay >40%), where high clay content corresponds to high ^137^Cs activity^26^.

##### Vertical distribution of ^137^Cs

**Figure 7:**
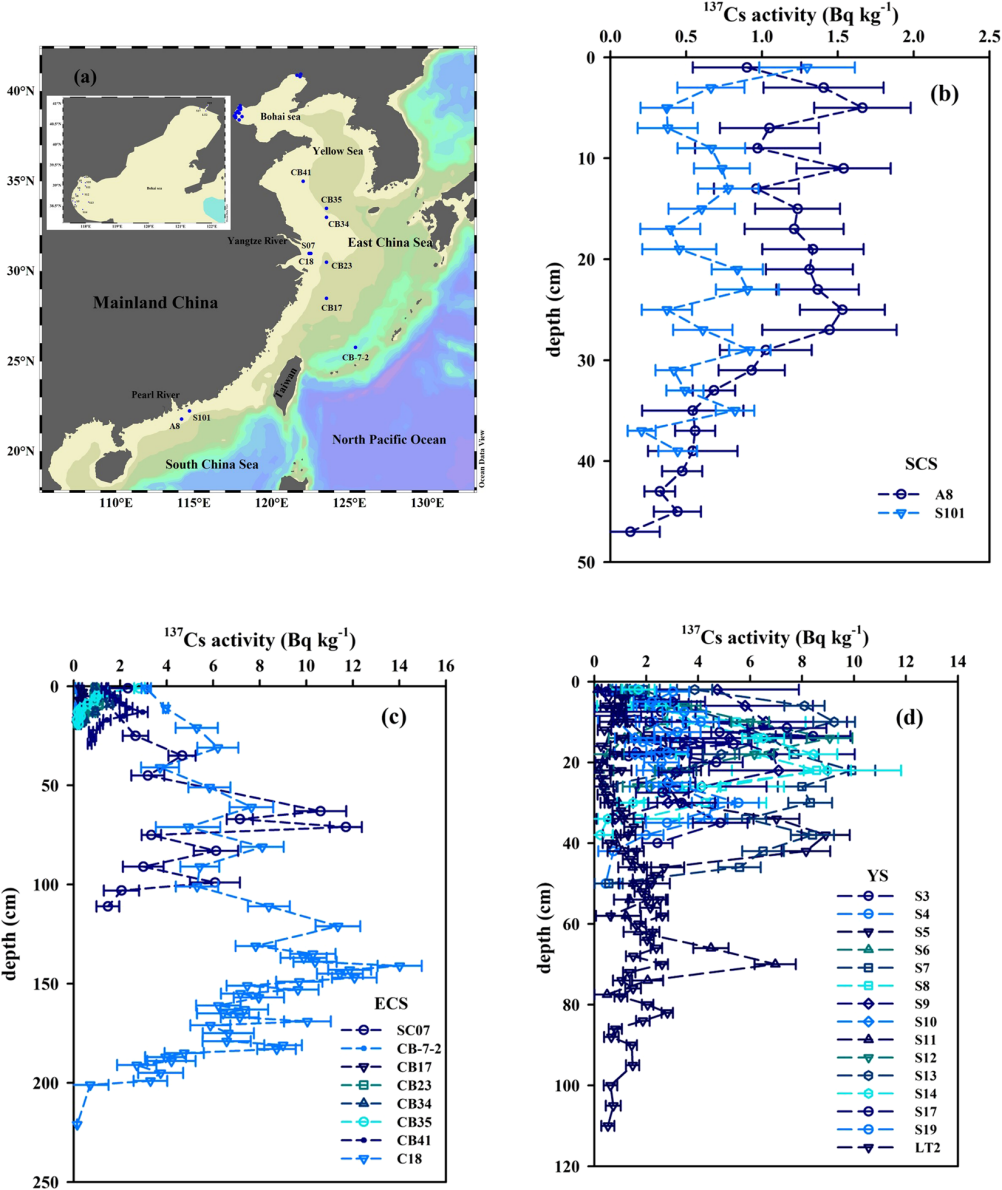
Sampling sites for analyzing ^137^Cs in sediment cores from the China Seas (**a**) and its vertical profiles in the South China Sea (**b**), East China Sea (**c**) and Yellow Sea (**d**). The sediment ^137^Cs data are cited from previous studies^5,6,29,31,44,45^. This figure was prepared with the free software Ocean Data View (ODV 5.1.2) (Schlitzer, R., Ocean Data View, https://odv.awi.de, 2018) (a) and Sigma–Plot professional 10.0 software (b–d).

The ^137^Cs activity in 25 sediment cores of the China Seas was collected from published papers (ECS–8 cores^5,31,44,45^; SCS–2 cores^6^; YS–15 cores^29^) and their profile patterns are plotted in Fig. 7.

The activity level of ^137^Cs in the different sediment cores displays huge spatial variability with distance from the shore. In the nearshore, the ^137^Cs activities show large fluctuations with respect to core depth (e.g., the coastal YS; Fig. 7d). In the shelf zone, the ^137^Cs activity is well preserved in the sediment cores (e.g., the SCS and ECS shelf: Fig. 7b,c). The vertical distribution of ^137^Cs in the shelf zone shows an initial increase down core, a prominent maximum appears in the mid–layer, and then a marked decline further down core. Indeed, the variation of ^137^Cs activity with depth in well preserved sediment cores usually reflects the input and depositional history of ^137^Cs, which is widely used to reconstruct sedimentary chronology in the marine environment^6,44,45^. For example, utilizing the time marker of ^137^Cs fallout pulse input (i.e., the ^137^Cs peak concentration in sediment cores corresponding to the global fallout maximum, circa 1963), Wu *et al*.^6^ calculated the sedimentation rate in the northern SCS shelf to be ~0.328 cm yr^−1^, which agrees with the rate calculated by an another independent natural radionuclide–^210^Pb_ex_ (~0.337 cm yr^−1^). This indicates that biological perturbation in the northern SCS shelf is limited^5^. Therefore, the temporal change of ^137^Cs input in the sediment core could be reflected by the sedimentary record.

**Figure 8:**
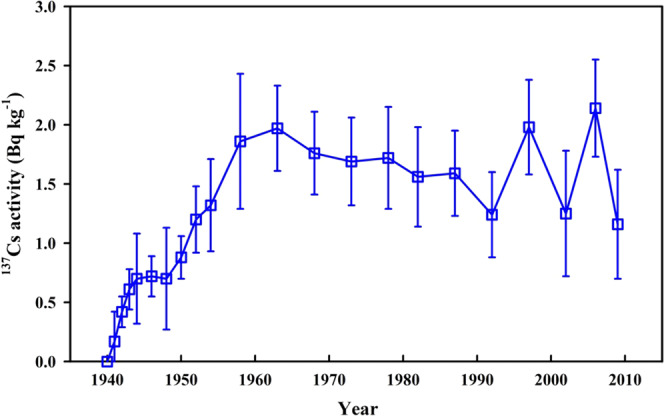
Temporal variations of ^137^Cs activity in sediment core A8 from the northern South China Sea shelf. This figure was prepared with Sigma–Plot professional 10.0 software.

Here, we discuss the temporal variation of ^137^Cs in the northern SCS shelf (Fig. 8). It is of note that the influence of diffusion and mixing generally exists, although the dominant process on the shelf is sedimentation. Therefore, we cannot present the annual change of ^137^Cs. Nevertheless, we can discuss the source influences and the features of ^137^Cs on a ten–year timescale by subdividing the sedimentary record into four time periods: pre–1945, 1946–1965, 1965–1985, and post–1986. Pre–1945, there was no the input of anthropogenic radionuclide ^137^Cs into the earth’s environment. However, the ^137^Cs signal was traced in depths of the sediment core A8 corresponding to this period, which is possibly caused by the post depositional downcore mixing of ^137^Cs originating from the global fallout and the Pacific Proving Grounds (PPG) tests conducted in the early 1950s. Between 1946–1965, the ^137^Cs activity dramatically increased because of large–scale atmospheric nuclear weapon testing and test in the PPG. The highest ^137^Cs activity (1.97 ± 0.36 Bq kg^−1^) in 1960–1965 indicates maximum ^137^Cs deposition by global fallout in 1963, which is often used as a time marker for the study of sedimentary chronology. From 1965 to 1985, the ^137^Cs activities showed a slight decrease from 1.76 to 1.56 Bq kg^−1^. This decrease was not as sharp as expected as the global large scale atmospheric nuclear weapon testing had been banned in this period. This suggests a continuous input of ^137^Cs derived from the PPG via the North Equatorial Current and Kuroshio transport. Post–1986, the ^137^Cs activities showed a gradual decrease, which was inconsistent with the termination of atmospheric nuclear weapon testing during this period. The reason is similar as in the above discussion during the period of 1965–1985.

**Figure 9:**
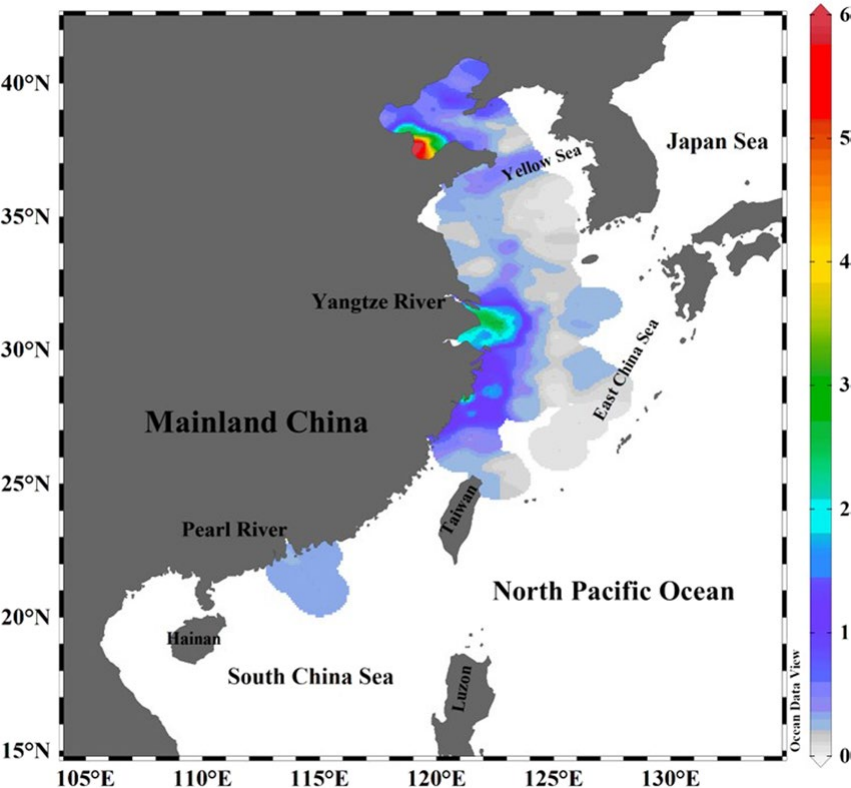
The lateral distribution of apparent sedimentation rates (cm yr^−1^) in the China Seas. The sediment rate datasets are cited from previous studies^5,6,29,31,44,45^. This map was drawn using the free software Ocean Data View (ODV 5.1.2) (Schlitzer, R., Ocean Data View, https://odv.awi.de, 2018).

Based on the above method (using the ^137^Cs time marker), the sedimentation rates in the ECS and the YS were also calculated. They ranged from 0.01 to 6.3 cm yr^−1^ (mean of 0.91 cm yr^−1^), which agreed with the results estimated with ^210^Pb_ex_^26,44,53,54^. The lateral distribution of apparent sedimentation rates in the China Seas is plotted in Fig. 9. Overall, high sedimentation rates appeared in the river mouth and nearshore, and gradually decreased with increasing distance from the shoreline. For example, the very high apparent sedimentation rates observed in the estuary of Yellow River and Yangtze River can be attributed to large amounts of terrestrial particles discharged from the two major rivers. In the shelf, low sedimentation rates suggest the sedimentary process is dominant. In contrast, in the nearshore, riverine input of terrestrial particles is a major influencing factor on the sedimentation rates. For example, the distribution of sedimentation rates decreased southwards along the inner shelf and offshore, which is consistent with the dispersal of CDW carried terrestrial particles. The apparent sedimentation rates in the YS showed an increasing trend from east to west. Therefore, ^137^Cs was a great tracer for sedimentary chronology.

### The inventory of ^137^Cs

**Figure 10:**
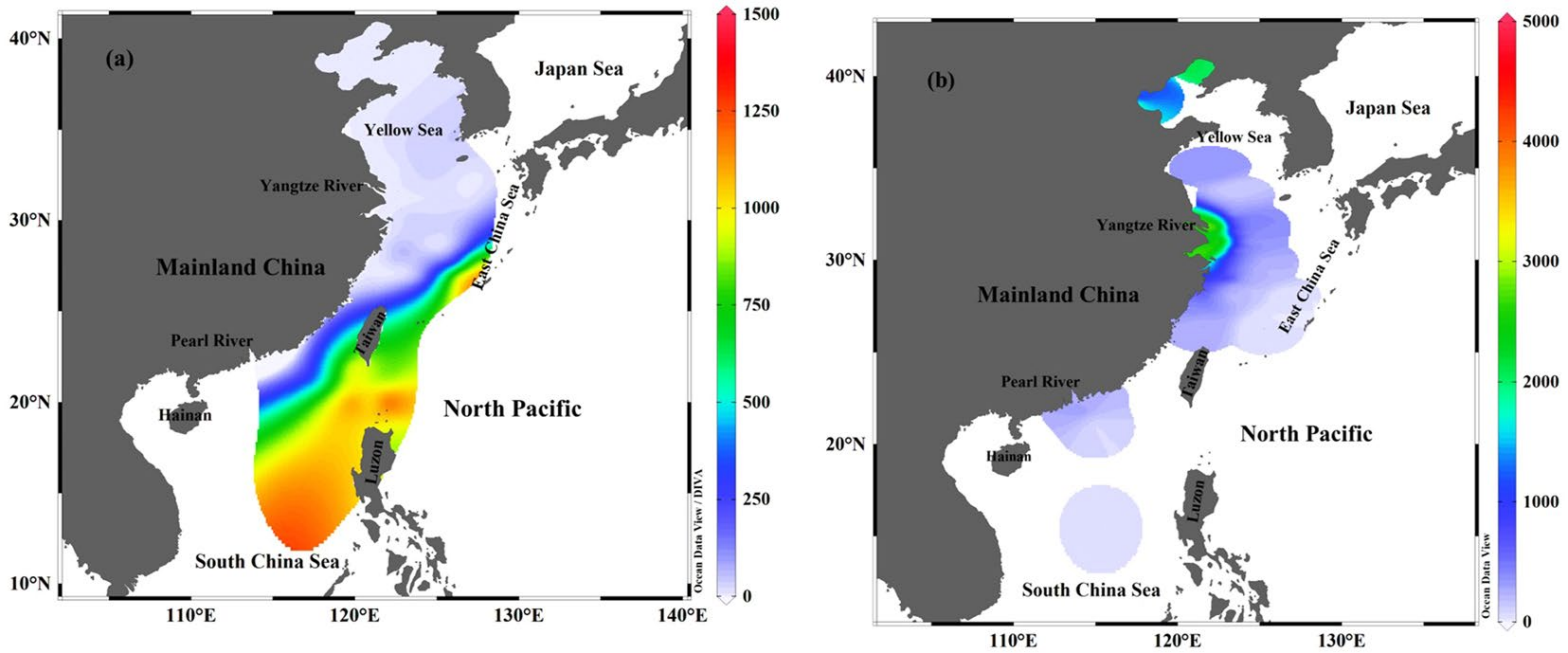
The distribution of ^137^Cs inventories (Bq m^−2^) in seawater (**a**) and sediment (**b**) of the China Seas. ^137^Cs inventories are cited from previous studies^5,6,14,24,26,28,29,31,45^. Note that, the ^137^Cs inventory in sediment core of the SCS basin is calculated based on ^239+240^Pu inventory and ^137^Cs/^239+240^Pu activity ratio. This map was drawn using the free software Ocean Data View (ODV 5.1.2) (Schlitzer, R., Ocean Data View, https://odv.awi.de, 2018).

The inventory of ^137^Cs in the water column and the sediment cores is calculated by integrating the activity measured at each depth^24,55^. The ^137^Cs inventory of seawater and sediment in the China Seas was reviewed and their spatial distributions are plotted in Fig. 10a,b, respectively.

In the ECS water column, the ^137^Cs inventory varied from 3 to 908 Bq m^−2^, averaging 88 Bq m^−2^, which is lower than that expected solely from global fallout within the same latitudinal zone (20°–35°N: ~750 Bq m^−2^)^56^. Indeed, the quantity was lower than that of the Atlantic Ocean (~1190 Bq m^−2^, 20°–35°N) and the Pacific Ocean (~1440 Bq m^−2^, 20°–35°N)^3^. The low ^137^Cs inventory was potentially due to the shorter turnover time of ESC water and rapid scavenging of ^137^Cs compared with the open ocean. The water turnover time of ECS is estimated to be less than 3 months^24^, which makes preserving ^137^Cs in the water column unfavorable in the ECS. Additionally, a large number of terrestrial particles discharged from the Yangtze River and the enhanced biomass in the ECS^57,58^ create favorable conditions for ^137^Cs scavenging in the water column^59^. Therefore, the bulk of ^137^Cs is preferentially deposited into sediments, resulting in the high ^137^Cs inventory of the ECS sediment^5,31,45^. For example, the ^137^Cs inventory in the ECS sediment exhibited a wide range of 39–3732 Bq m^−2^ (average = 970 Bq m^−2^), which is over twice what is predicted solely from global fallout (~750 Bq m^−2^)^56^. The distribution of ^137^Cs inventory in the ECS sediment decreased significantly away from the shore, indicating that the input of terrestrial particle is an important ^137^Cs source (see in discussion below). High ^137^Cs inventory also indicate elevated recent deposition of particles near the estuary, most likely, preferential deposition and accumulation. The distribution of apparent accumulation rates in the China Seas indicates a high sedimentation rate near the Yangtze River estuary with the value reaching up to 4 cm yr^−1^, which gradually decreased with distance from the shoreline^5,53^. Overall, the distribution of ^137^Cs inventory in the sediment of ECS is in agreement with the distribution of sedimentation rates.

It is worth noting that the reported ^137^Cs inventory in the upper 600 m in the northeastern SCS and the Luzon Strait (0–600 m) represents only a part of the whole water column^28^. We thus carefully examined the literature data concerning the profiles of the ^137^Cs activity in the Pacific Ocean and found that the spatial variation is limited where the typical percentage of the ^137^Cs inventory at a depth interval of 0–600 m accounted for 62.9–76.5% (average of 69.4%, n = 36) of the whole water column^3,4,11,39^. Given that the depth profiles in the northeastern SCS in the upper 600 m were rather similar to the Pacific, we extrapolated the upper 600 m inventory to the whole water column using the percentage partitioning in the Pacific Ocean. Accordingly, the ^137^Cs inventory in the northeastern SCS water column was calculated, varying from 49 to 1257 Bq m^−2^ (average = 867 Bq m^−2^)^28,39^, which is over two times what is predicted solely from global fallout (~412 Bq m^−2^, 10–20°N)^56^. This ^137^Cs inventory is significantly higher than that found in the ECS (~88 Bq m^−2^). Note that, in the northeastern SCS, the ^137^Cs inventory in the water column was higher than that found in the sediment core (48–401 Bq m^−2^, averaging 186 Bq m^−2^), which is different from the ECS. This indicates that, in the deep marginal sea (e.g., SCS), ^137^Cs largely resides in the water column, in contrast to the ECS where ^137^Cs is preferentially stored in the sediment. Therefore, for the deeper marginal sea or open ocean the ^137^Cs inventory of the water column positively correlates with water depth, and the ^137^Cs inventory of sediment cores negatively correlates with water depth.

The ^137^Cs inventory in the YS water column varied from 0.3 to 36 Bq m^−2^ (average = 7 Bq m^−2^)^26^, which is three orders of magnitude lower than that expected solely from global fallout within the same latitudinal zone (30°–50°N: ~1016 Bq m^−2^)^56^. The reason for the low ^137^Cs inventory in the YS water column is similar to that of the ECS, and depends on the input of terrestrial particles, water depth, the turnover time and quick of scavenging ^137^Cs as discussed above. Accordingly, this would lead to higher ^137^Cs inventory preserved in the YS sediment cores. The ^137^Cs inventory of the YS sediment exhibited a wide range, from 240 to 5071 Bq m^−2^, averaging 1736 Bq m^−2^ ^26,29^, which is nearly twice that expected solely from global fallout within the same latitudinal zone (30°–50°N: ~1016 Bq m^−2^)^56^. This ^137^Cs inventory is also higher than those calculated for the ECS and the SCS, indicating the ^137^Cs carried by the terrestrial particles is more quickly and easily scavenged and deposited into the sediment.

### Mean residence time of ^137^Cs in the China Seas

The effective environmental half–life of ^137^Cs is a good indicator for evaluating the environmental risk of ^137^Cs in the China Seas. In the marine environment, ^137^Cs is a good tracer to study water mass transport due to its high solubility in seawater^2^. In marine systems, the distribution coefficient (*K*_*d*_) of ^137^Cs in sediment is reported to be ~2000, and ^137^Cs scavenging from the water column mainly depends on its diffusion and decay rate^60^. In general, ^137^Cs activity in surface seawater decreases exponentially with time. The temporal change of ^137^Cs activity in the ocean can be expressed by the following exponential function^61^,

1 $${R}_{t}={R}_{0}\exp (-at)$$

where *R*_*t*_ is the ^137^Cs activity during year *t* and *R*_0_ is the ^137^Cs activity at *t* = 1967.

**Figure 11:**
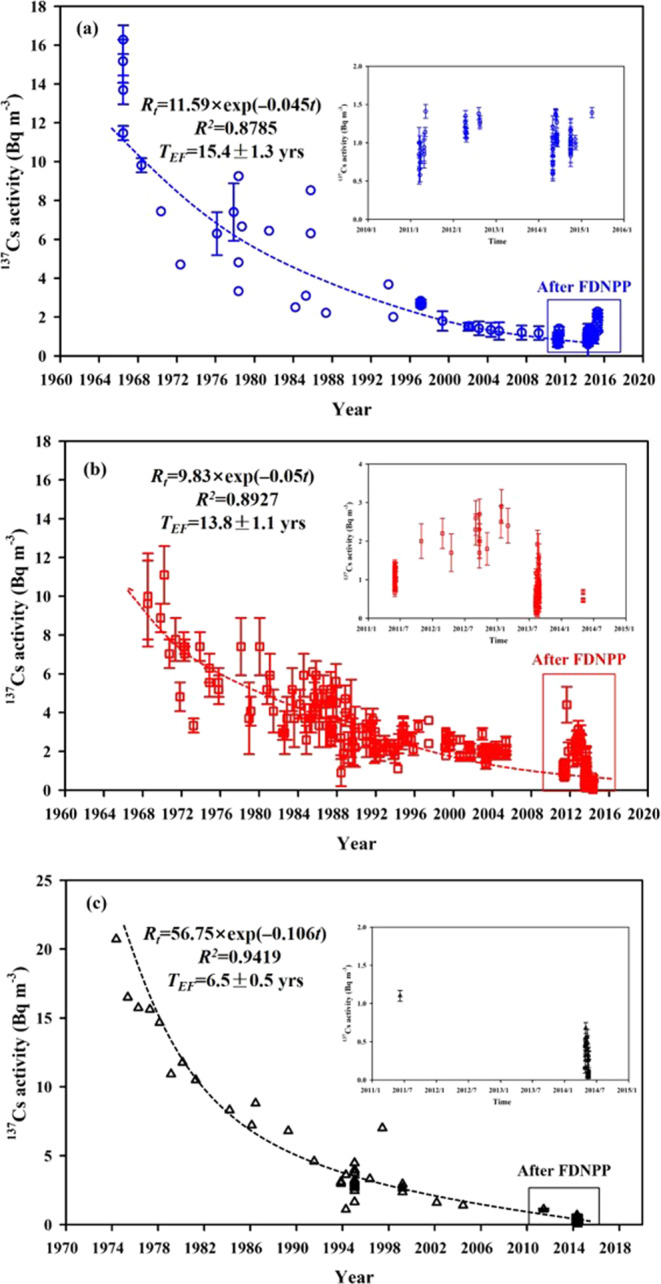
Temporal variations of surface ^137^Cs activity in the South China Sea (**a**), East China Sea (**b**) and Yellow Sea (**c**) over the 1960–2020. ^137^Cs data are cited from previously published studies^12,13,24–28,31^ and the IAEA’s marine information system (MARIS) (https://maris.iaea.org/). This figure was prepared with Sigma–Plot professional 10.0 software.

A previous study roughly estimated the effective environmental half–life of ^137^Cs (*T*_*EF*_) in the SCS and the ECS based on limited *in situ* ^137^Cs data^13^. Here, the most comprehensive ^137^Cs dataset in the China Seas was collected in order to further improve the accuracy or reduce the uncertainty of this estimate. The temporal change of ^137^Cs activity in the China Seas surface seawater over the past 60 years, by expanding our dataset to include ^137^Cs datasets from previous studies^12,13,25,26,28,31^, is shown in Fig. 11a–c (SCS: Fig. 11a, ECS: Fig. 11b, YS: Fig. 11c). According to this expanded ^137^Cs dataset, fitted equations of ^137^Cs activity with respect to time are shown in Fig. 11a–c. The *T*_*EF*_ in the China Seas was then determined as 15.4 ± 1.3 years for the SCS, 13.8 ± 1.1 years for the ECS, and 6.5 ± 0.5 years for the YS. These estimated values are slightly lower than previous results based on the more limited ^137^Cs datasets^13,26^. The longer *T*_*EF*_ in the SCS indicates that a large amount of ^137^Cs is preserved in the water column, which is consistent with the higher ^137^Cs inventories found in the SCS compared to the ECS and YS. Our estimates were also lower than those calculated in the WNP at the same latitude (15–24 years)^61^ and in the coastal water of Japan (~18.7 years)^62^. Additionally, the estimated *T*_*EF*_ values in the China Seas were significantly lower than the ^137^Cs half–life. *T*_*EF*_ is related to the natural decay of ^137^Cs and subsequent marine processes, including vertical and horizontal water mass movements and particle scavenging. Note that estimated values are usually larger than the real values since the fraction of radioactive decay and scavenging activity are ignored. Nevertheless, they are indicative of current fallout deposition and terrestrial inputs, although the upper limit of ^137^Cs mean residence time (*T*_*M*_) is a more realistic indicator^62^. The *T*_*M*_ could be expressed using the following formula^62^,

2 $$\frac{1}{{T}_{M}}=\frac{\mathrm{ln}(2)}{{T}_{EF}}-\frac{\mathrm{ln}(2)}{{T}_{R}}$$

where *T*_*EF*_ is the effective environmental half–life and *T*_*R*_ represents the radiological half–life (*T*_*R*_ = 30.17 years). Using Eq. (2), *T*_*M*_ in the China Seas was calculated as 45.6 ± 3.8 years for the SCS, 36.8 ± 3.1 years for the ECS, and 12.0 ± 1.0 years for the YS. Our estimated residence times will help to understanding the turnover time of ^137^Cs in the China Seas. Longer residence times indicate that ^137^Cs activity decreases more slowly with time. For example, the ^137^Cs residence time of SCS is longer than those in the ECS and the YS, indicating the activity and inventory of ^137^Cs in the former is higher than the latter, which agrees with the above field observation results. In the future, the activity level of ^137^Cs in the SCS would be still higher than that observed in the ECS and the YS with the assumption of no additional input of ^137^Cs. Our estimated residence times of ^137^Cs in the China Seas are slightly lower than those obtained in the Atlantic Ocean (~100 years)^63^ and the coastal waters of Japan (~60–70 years)^62^.

### Budget of ^137^Cs in the China Seas

The activity level of ^137^Cs in water column of the China Seas depends on its sources and sinks. The input sources of ^137^Cs in the China Seas mainly include discharge from the Chinese nuclear power plants, the input by FDNPP, riverine input, direct deposition of global fallout and exchange with the Pacific Ocean. The output of ^137^Cs in the China Seas includes radioactive decay and burial. Until now, there have been no reports of ^137^Cs leakage from Chinese nuclear power plants in the China Seas. Previous studies confirmed the influence of FDNPP in the China Seas was minor^13,24,26^.

The temporal variation of ^137^Cs inventory in the water column of the China Seas is expressed by the following equation:

3 $$\frac{dW}{dt}={R}_{{137}_{Cs}}+{A}_{{137}_{Cs}}+{O}_{{137}_{Cs}}-{S}_{{137}_{Cs}}-\lambda \times W$$

where *W* represents the total ^137^Cs inventory in the water column (Bq), λ (0.023 yr^−1^) is the ^137^Cs decay coefficient. The last term of −*λW* in the right–hand of the equation is ignored when the decay–corrected ^137^Cs concentration was used to calculate *W* values. Here, all the ^137^Cs data is decay–corrected to January 1, 2020. *R*_137*Cs*_, *A*_137*Cs*_, *O*_137*Cs*_, *S*_137*Cs*_ represent the riverine input flux of ^137^Cs (Bq yr^−1^), the ^137^Cs flux of global fallout (Bq yr^−1^), the net ^137^Cs exchange flux with the North Pacific and the ^137^Cs burial flux in surface sediment (Bq yr^−1^), respectively. With the assumption that the China Seas are in steady state, the variation of ^137^Cs should keep constant over time (i.e., *dW*/*dt* = 0). However, as discussed above, the temporal change of ^137^Cs activity in surface water showed an exponential decrease. Here, based on the distribution of ^137^Cs inventories in the water column (taking an average value of ^137^Cs inventories) and the surface area (as calculated in Google Earth) of the China Seas (detail in Table S3 in the SI), we calculate total water column ^137^Cs inventories of (28.05 ± 5.32) × 10^14^ Bq for the SCS, (1.40 ± 0.90) × 10^14^ Bq for the ECS, and (6.98 ± 5.34) × 10^12^ Bq for the YS. Using the above calculated mean residence time of ^137^Cs in the China Seas, the ^137^Cs fluxes in the water column are (6.15 ± 1.16) ×10^13^ Bq yr^−1^ for the SCS, (3.80 ± 2.45) × 10^12^ Bq yr^−1^ for the ECS, and (5.82 ± 4.45) ×10^11^ Bq yr^−1^ for the YS. Therefore, the total ^137^Cs flux in the water column of the China Seas is calculated to be (65.88 ± 14.50) × 10^12^ Bq yr^−1^.

The burial flux of ^137^Cs in sediment depends on the ^137^Cs activity, sedimentation rate and bulk density. Zhang *et al*.^26^ estimated the burial fluxes of ^137^Cs as 7.3 ± 2.3 Bq m^−2^ yr^−1^ for the ECS, 6.9 ± 1.6 Bq m^−2^ yr^−1^ for the YS and 22.4 ± 8.7 Bq m^−2^ yr^−1^ for the Bohai sea. Based upon their work, we use an expanded ^137^Cs dataset to further estimate the burial flux of ^137^Cs in the whole China Seas and determined fluxes of (5.62 ± 1.77) × 10^12^ Bq yr^−1^ for the ECS, (4.49 ± 1.31) × 10^12^ Bq yr^−1^ for the YS (including Bohai sea: (1.73 ± 0.67) × 10^12^ Bq yr^−1^). Note that previously there was no reported ^137^Cs inventory in the SCS basin. Nevertheless, based on the ^239+240^Pu inventory (~3.75 Bq m^−2^)^64^ in the SCS basin and the ^239+240^Pu/^137^Cs activity ratio of global fallout (0.0285 ± 0.0038, decay–corrected to January 1, 2020)^34^, we calculated the ^137^Cs inventory in the SCS basin to be ~75 Bq m^−2^, assuming a similar behavior between ^137^Cs and ^239+240^Pu. On the SCS shelf, the ^137^Cs inventory was estimated to be 207.9 ± 133.8 Bq m^−2^ (see details in Table S4 in the SI)^6^. The total inventory of ^137^Cs was thus calculated to be (3.96 ± 1.34) ×10^14^ Bq for the SCS. According to the distribution of sedimentation rates in the SCS, the burial flux of ^137^Cs was further calculated to be (6.95 ± 2.35) ×10^12^ Bq yr^−1^ for the SCS. The total burial flux of ^137^Cs in the China Seas was calculated to be (17.06 ± 5.43) ×10^12^ Bq yr^−1^. We point out that these estimates are subject to large uncertainties due to the limited collection of water column and sediment field data.

There are many rivers discharging a large quantity of terrestrial particulates into the China Seas as a result of soil erosion in the river drainage area. Riverine input is thus an important ^137^Cs source to the China Seas. Previous studies suggest the input of ^137^Cs from the Yangtze River and Yellow River is a major source to the ECS and YS, respectively^5,26^. The precise estimate of the total riverine input of ^137^Cs to the China Seas needs a large number of *in–situ* ^137^Cs data for each river. However, this is an extensive work to carry out, and currently only very limited ^137^Cs data in Chinese rivers are available. Here, we highlight the three major rivers in China: namely, the Yangtze River, Pearl River and Yellow River. The following equation can be used to roughly estimate the riverine input of ^137^Cs to estuaries with large drainage basin to estuarine area ratios^65,66^, and has been successfully applied in the ECS and the YS^5,26^.

4 $${I}_{d}={A}_{d}\times {I}_{f}\times {f}_{e}$$

where *A*_*d*_ is the area of the drainage basin, *I*_*f*_ is the ^137^Cs inventory in the soil of the river drainage basin and *f*_*e*_ is the fraction of ^137^Cs inventory eroded each year from the watershed (*f*_*e*_ = ln2/residence time of ^137^Cs in the watershed). The collective inventory of ^137^Cs in soil cores from river drainage basins around the China Seas are shown in Table S5 (SI). The average inventory of ^137^Cs was calculated based on the three major river systems flowing into the SCS (Pearl River), the ECS (Yangtze River) and the YS (Yellow River). The residence times of ^137^Cs in various global river drainage basins vary greatly, ranging from 800 to 4100 years^5,26,67–69^. Considering that the drainage area of the Yangtze River is among the largest in the world, taking the upper limit of residence time (4100 years) is reasonable^26^. The residence time of ^137^Cs in other rivers is shown in Table S6 (SI). Accordingly, the calculated riverine input of ^137^Cs is (1.66 ± 0.63) × 10^11^ Bq yr^−1^ for the SCS, (5.01 ± 0.83) × 10^11^ Bq yr^−1^ for the ECS, and (5.82 ± 1.72) × 10^11^ Bq yr^−1^ for the YS, resulting in a total riverine input of ^137^Cs into the China Seas of (12.49 ± 3.18) ×10^11^ Bq yr^−1^. The input of ^137^Cs discharged from the three major rivers (Yangtze River, Yellow River and Pearl River) is calculated to be (7.35 ± 1.69) ×10^11^ Bq yr^−1^, accounting for ~60% of the riverine input to the China Seas. These three major rivers account for ~70% of the terrestrial particulate matter discharged into the China Seas^70–73^, which is slightly higher than the ^137^Cs fraction contributed by riverine inputs (~60%).

It is well known that the large–scale global atmospheric nuclear weapons testing conducted in the 1950s and the early 1960s resulted in the worldwide deposition of ^137^Cs. The peak ^137^Cs deposition flux appeared in 1963^74^, and then gradually decreased with time (with the exception of the additional depositional influence of the Chernobyl nuclear accident in 1986) due to the global ban of atmospheric nuclear weapons testing. At present, the ^137^Cs deposition of global fallout in the China Seas is mainly originating from the resuspension and transport of East Asian dust packaged ^137^Cs^74^. Through long–time series observation at the Japan Meteorological Station (36.05° N, 140.13° E), the deposition flux of ^137^Cs is calculated to be 0.22 ± 0.09 Bq m^−2^ yr^−1 74^. At another observation station in Shanghai (31.23° N, 121.40° E), the deposition flux of ^137^Cs is estimated to be 0.33 ± 0.20 Bq m^−2^ yr^−1 49^. For this study, we used the mean value (0.28 ± 0.08 Bq m^−2^ yr^−1^) at the two observation stations as the deposition flux of ^137^Cs in the China Seas, as the China Seas are located between them. Then we calculated the direct ^137^Cs deposition of global fallout to be (9.80 ± 2.80) × 10^11^ Bq yr^−1^ for the SCS, (2.16 ± 0.62) × 10^11^ Bq yr^−1^ for the ECS, and (1.34 ± 0.38) × 10^11^ Bq yr^−1^ for the YS. Our calculated result in the ECS is higher than the previously reported value^26^, since our study area is significantly larger than their area. The total ^137^Cs deposition flux of global fallout in the China Seas is calculated to be (13.30 ± 3.80) ×10^11^ Bq yr^−1^. Finally, according to the Eq. (3), the net ^137^Cs flux exchange between the China Seas and North Pacific is ~8.04 × 10^13^ Bq yr^−1^. The total ^137^Cs inventory in the China Seas is roughly estimated to be 5.4 × 10^15^ Bq, which is less than 1.0% of the ^137^Cs inventory in the global ocean. The ^137^Cs contribution to the China Seas from the oceanic input is estimated to be about 96.9%: the dominant ^137^Cs source. This result is consistent with the above discussion.

Overall, the total ^137^Cs inventories in water column and sediment core of China Seas are calculated to be (29.5 ± 6.3) × 10^14^ Bq and (17.6 ± 12.4) × 10^14^ Bq (data see in Tables S3 and S4 in the SI), accounting for 62.6% and 37.4%, respectively. In detail, the total ^137^Cs inventories in water column and sediment of the SCS are calculated to be (28.05 ± 5.32) × 10^14^ Bq and (3.96 ± 1.34) × 10^14^ Bq (data see in Tables S3 and S4 in the SI), respectively, accounting for 87.6% and 12.4%. This indicates that most of the ^137^Cs is well preserved in the SCS water column. In contrast, the percentage of the ^137^Cs inventory in water column and sediment of the ECS are about 20.6% and 79.4%, respectively, suggesting most ^137^Cs is deposited in the sediment. In the YS, the water column and sediments contain approximately 0.8% and 99.2% of the ^137^Cs inventory, respectively, suggesting that most of it is deposited in the sediment. Therefore, the bulk of ^137^Cs remains in the SCS water column, in contrast to the ECS and the YS where most of ^137^Cs is deposited in the sediments.

Additional work is needed to fully understand radio–cesium biogeochemistry and its fate in the environment. Accurate determination of radio–cesium isotopic composition could further help to identify its source; the ^134^Cs/^137^Cs isotopic ratio is widely used. However, this ratio is unavailable in the China Seas due to the decay of ^134^Cs to undetectable levels because of its short half–life (~2.06 years) and the absence of recent inputs. The ^135^Cs/^137^Cs isotopic ratio is a potential alternative chronometer–tracer to investigate ^137^Cs source contributions in the marine environment^75^. ^135^Cs is difficult to measure, complicating the study of its transport and fluctuations in the ocean. Therefore, developing methods for the determination of ^135^Cs and the ^135^Cs/^137^Cs isotopic ratio in the China Seas is of future research interest, which may help assess the environmental risk of Chinese nuclear power plants in the future. Lastly, understanding the biological speciation and transformation processes of ^137^Cs would also be useful for accurately evaluating its ecological impact.

## Supplementary information

Supplementary Information.
